# Development of coronary artery disease in patients with initially normal coronary arteries in the SCOT-HEART trial

**DOI:** 10.1007/s00330-026-12353-6

**Published:** 2026-03-05

**Authors:** Lia Avigdor, Steven E. Williams, Alan Ranieri Guimaraes, Kayleigh Wood, Jenny Ramsay, Phyo H. Khaing, Krystalina Sim, Giles Roditi, Nicholas L. Mills, Marc R. Dweck, David E. Newby, Michelle C. Williams

**Affiliations:** 1 British Heart Foundation Centre of Research Excellence, The University of Edinburgh, Edinburgh, UK; 2 University of Glasgow, Glasgow, UK

**Keywords:** Coronary artery disease, Multidetector computed tomography, Computed tomography angiography, Atherosclerosis

## Abstract

**Objectives:**

Individuals with normal coronary arteries may develop coronary artery disease (CAD). Coronary computed tomography (CT) angiography (CCTA) offers a non-invasive method to assess the development of CAD.

**Materials and methods:**

In a post-hoc observational study of the Scottish Computed Tomography of the HEART (SCOT-HEART) trial, we identified patients with normal coronary arteries on initial CCTA who subsequently underwent clinically indicated CT. Images were visually assessed for the presence, severity, and type of CAD.

**Results:**

Normal coronary arteries on baseline CCTA were present in 524 patients (mean age 53 ± 10 years, 38% male). After a median of 9.3 (Interquartile range, IQR: 9.3–10.8) years, 31 (6%) underwent repeat CCTA and 162 (31%) underwent chest CT. There were no differences in baseline clinical characteristics amongst those who did or did not have repeat CCTA, but those with subsequent chest CT were older and had higher cardiovascular risk scores. CAD was identified on 48% (*n* = 15) of CCTA and 25% (*n* = 41) of chest CT. Median time to CT scan on which CAD was identified was 8.1 (IQR: 6.9–9.7) years. There was no difference in all-cause mortality or combined CAD death or non-fatal myocardial infarction in patients who had CAD identified on subsequent CT. However, they were more likely to undergo invasive coronary angiography (adjusted hazard ratio [aHR] 4.94, 95% confidence interval [CI]: 1.95, 12.51; *p* < 0.001) and revascularization (aHR 19.99, 95% CI: 1.69, 237.1; *p* = 0.018), adjusted for age and sex.

**Conclusion:**

One third of patients with previously normal CCTA will develop CAD on clinically indicated CT imaging over a 10-year period.

**Key Points:**

***Question*** *In patients with normal coronary arteries on coronary computed tomography angiography (CCTA), the risk of developing CAD in the future is uncertain*.

***Findings*** *Among 524 patients with normal coronaries, CAD was identified on 48% of CCTA and 25% of chest CT during 10 years of follow-up*.

***Clinical relevance*** *A substantial proportion of patients with initially normal coronary arteries on CCTA later develop CAD, highlighting the need for clinicians to be alert for the development of new CAD in patients with initially normal coronary arteries*.

**Graphical Abstract:**

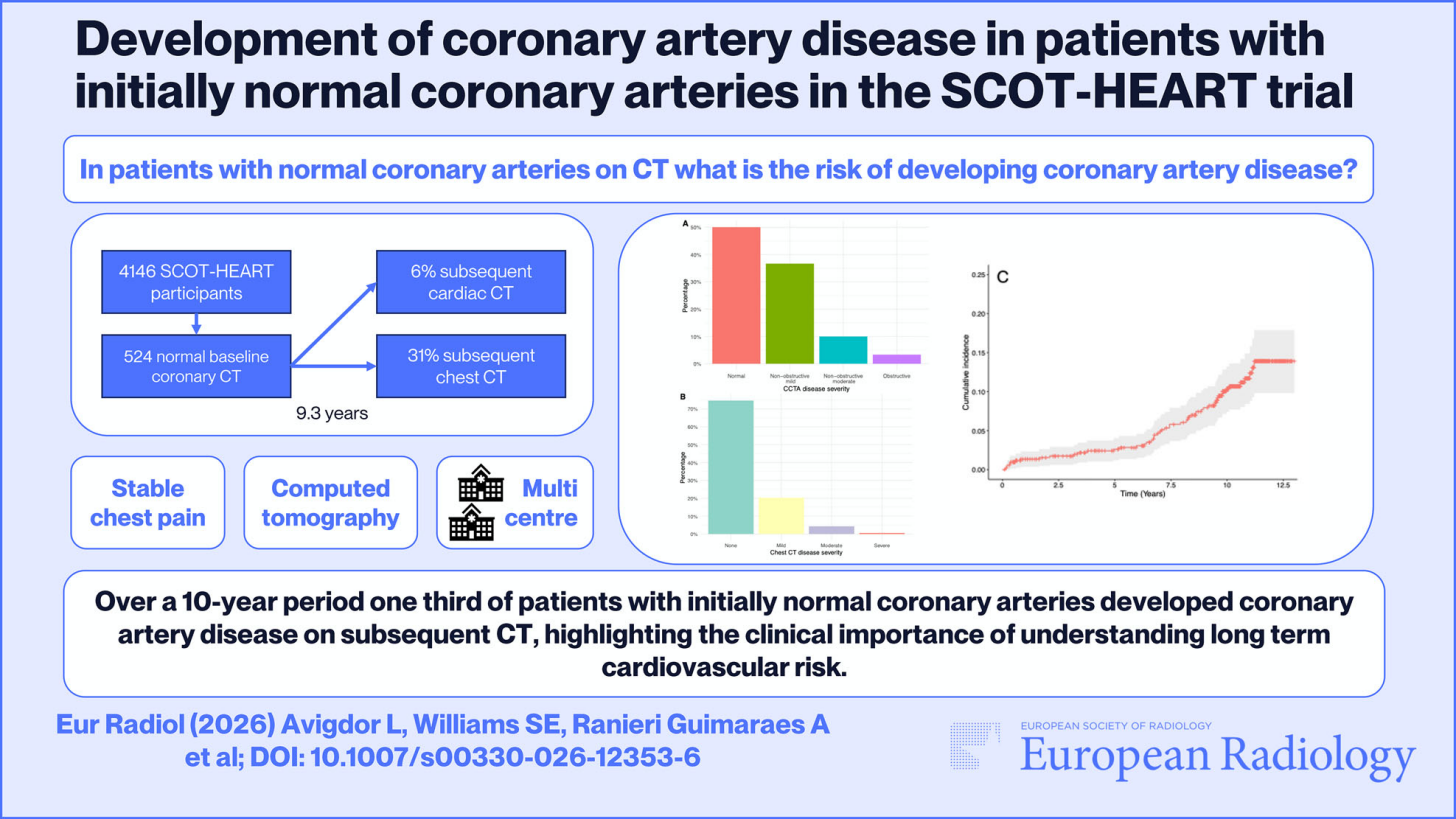

## Introduction

Patients with normal coronary arteries on coronary computed tomography angiography (CCTA) have a good long-term prognosis with a low risk of subsequent cardiovascular events [1]. However, some patients with initially normal coronary arteries will develop coronary artery disease (CAD). The factors associated with the development and progression of CAD remain incompletely understood. Determining which patients will develop CAD would enable the targeting of preventative medical therapies. In addition, understanding the timeline of the development of CAD could help determine the optimal time interval for follow-up imaging.

Numerous studies, including the landmark Framingham Heart Study, have identified a range of risk factors associated with the development of CAD. However, these cardiovascular risk factors do not fully explain why some individuals develop CAD while others do not. The pathophysiology of CAD is complex, involving a combination of lipid deposition, inflammation, endothelial dysfunction, fibrosis, and calcification. Previous registry studies have assessed clinical factors associated with the development of CAD on non-contrast CT for coronary artery calcium scoring [2, 3]. However, the development of incident CAD associated with the development of CAD on CCTA in patients with initially normal coronary arteries has not previously been assessed.

The Scottish Computed Tomography of the HEART (SCOT-HEART) trial established the utility of CCTA to guide the management of patients with stable chest pain [4–6]. In the SCOT-HEART trial, patients with normal coronary arteries on CCTA had a good long-term prognosis with a low risk of subsequent coronary events after 5 years [1]. However, during their routine clinical care, some patients will undergo repeat CCTA. In addition, non-gated chest CT may also be performed for the evaluation of other conditions, where the presence of coronary artery calcification would highlight the development of CAD. Evaluating the use of subsequent CT scans and the development of CAD on routinely performed imaging in patients in the SCOT-HEART trial, therefore, provides a valuable opportunity to understand the mechanisms of CAD.

This study aims to assess the use of subsequent CCTA or non-gated chest CT in patients with initially normal coronary arteries in the SCOT-HEART trial, and to assess the development of new CAD in these patients.

## Materials and methods

### Study design

This observational cohort study assessed the use of downstream CT imaging and the development of new CAD in patients with initially normal CCTA in the SCOT-HEART trial. SCOT-HEART is an open-label, multicenter randomized controlled trial that assessed the use of CT in patients who attended outpatient cardiology clinics with symptoms of suspected angina due to CAD. The primary outcomes of the SCOT-HEART trial have previously been published, but the use of subsequent CT has not been previously assessed [4–7]. The SCOT-HEART trial was performed with ethical approval, and participants provided written informed consent.

### Participants

In the SCOT-HEART trial, 4146 participants aged 18–75 years were recruited from cardiology clinics where they were referred for assessment of suspected angina due to CAD. Participants were randomized 1:1 to standard care or standard care plus CCTA. For the purposes of the present sub-study, we identified participants from the SCOT-HEART trial database who had normal coronary arteries on CCTA. We excluded patients with CAD on CCTA, a calcium score greater than zero, and those with a prior history of CAD.

### Baseline clinical information and cardiovascular outcomes

Baseline clinical information was obtained from the SCOT-HEART database. This included demographic information and cardiovascular risk factors. The 10-year cardiovascular risk score was assessed using the ASSIGN (assessing cardiovascular risk using SIGN guidelines) 10-year cardiovascular risk score [8]. Information on cardiovascular events, mortality, and revascularization at 10 years was obtained from the SCOT-HEART database based on nationally coded healthcare information from Public Health Scotland via the Electronic Data Research and Innovation Service.

### Identification of CAD on subsequent CT

The national picture archiving and communication system (PACS) of NHS Scotland was used to identify whether a participant had subsequently undergone cardiac CT (CCTA or non-contrast CT for coronary artery calcium scoring) or chest CT. Indication for CT imaging was obtained from the report text. Information on new diagnoses, changes in risk factors, and medication adherence during the 10 years of follow-up was not available for this analysis. Images of the subsequent CT scans were reviewed. The presence of CAD was assessed visually on CCTA by a trained observer. The severity of coronary artery stenoses was defined based on the luminal diameter as normal (< 10%), mild non-obstructive (10–49%), moderate non-obstructive (50–70%), or obstructive stenosis (> 70%) in one or more major epicardial vessels or > 50% stenosis in the left main stem. Coronary artery plaque was classified as calcified, non-calcified, or mixed plaque. On chest CT, the presence of coronary artery calcification was visually assessed and classified as mild, moderate, or severe [9].

### Statistical analysis

Statistical analysis was performed using R, version 4.3.2 (R Foundation for Statistical Computing). Quantitative data are presented as mean and standard deviation or median and interquartile range (IQR). Statistical significance was assessed using the Chi-squared test, Student's *t*-test, or Mann–Whitney *U*-test as appropriate. Clinical outcomes were assessed with Cox proportional hazard models and Kaplan–Meier plots, with hazard ratios (HR) provided with 95% confidence intervals (CI). Multivariable models were created and adjusted for age and sex. A two-tailed *p* value of < 0.05 was considered statistically significant.

## Results

### Study population

**Fig. 1:**
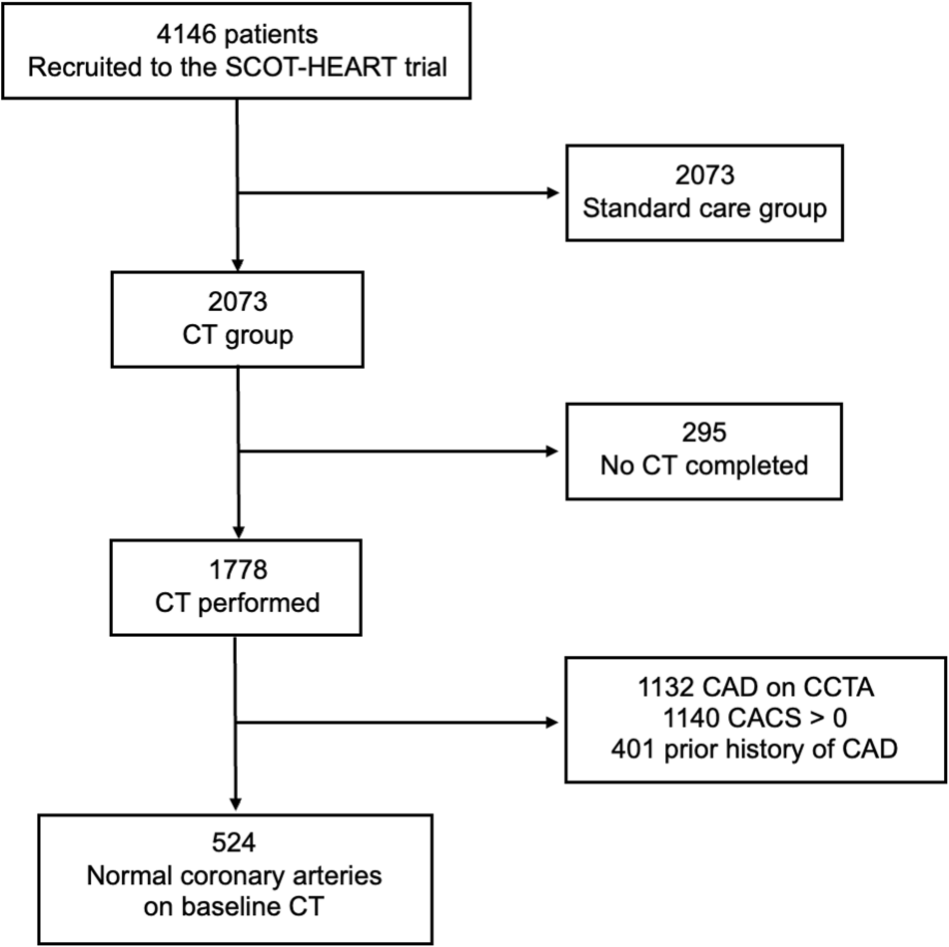
Profile of participants in the SCOT-HEART trial who were included in this analysis. CT, computed tomography

**Fig. 2:**
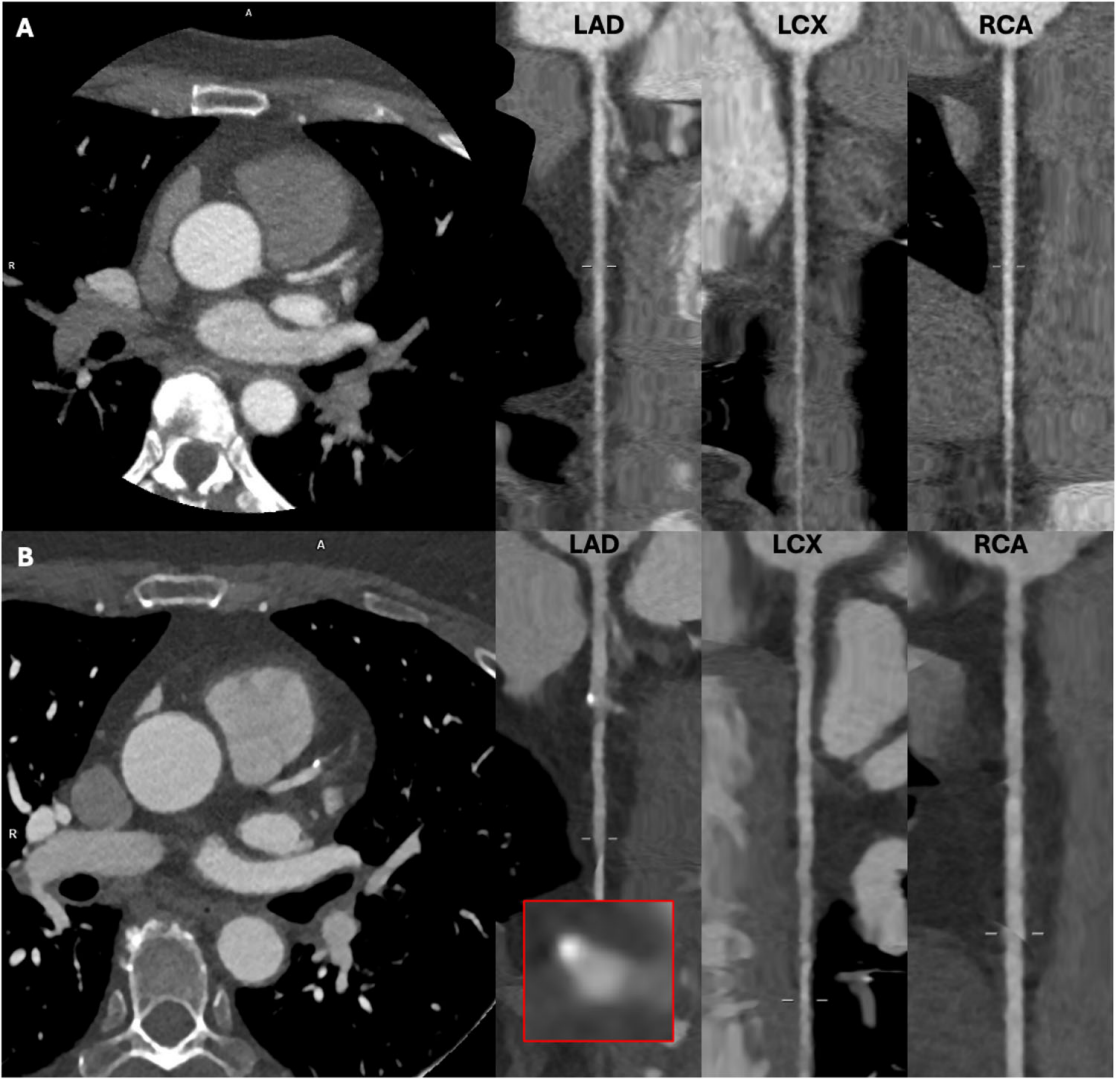
Development of CAD in a female patient, age 51 years, with a body mass index of 31 kg/m^2^, typical chest pain, and a family history of CAD. Baseline coronary CT angiogram (**A**) with axial image and curved planar reformations of the left anterior descending (LAD), left circumflex (LCx), and right coronary arteries (RCA) were normal. Coronary CT angiogram (**B**) 11 years later showed a calcified plaque in the mid LAD, demonstrated on the axial image, curved planar reformation, and a cross-sectional image (red box)

**Fig. 3:**
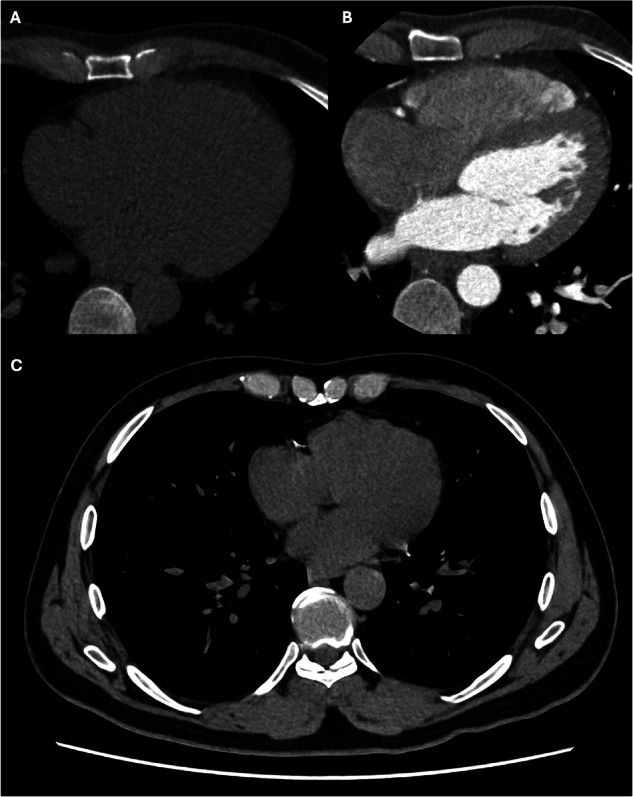
Development of CAD in a 43-year-old male smoker, with atypical chest pain, hypercholesterolemia, and a family history of CAD. At baseline, the coronary artery calcium score was zero (**A**), and the coronary CT angiogram was normal (**B**). Non-contrast CT chest (**C**) 10 years later showed calcified plaques in the right coronary artery and left circumflex coronary artery

Of the 2073 patients allocated to CCTA in the SCOT-HEART trial, 1778 had CCTA performed. Of these 524 had no CAD on CCTA, a coronary artery calcium score of zero, and no history of previous CAD (Fig. 1). Patients with normal coronary arteries had a mean age of 53 ± 10 years, 38% were male (*n* = 263), and they had a low frequency of cardiovascular risk factors (Table 1). Of the 524 patients, 6% (*n* = 31) had a subsequent cardiac CT (Fig. 2) and 31% (*n* = 162) had a subsequent chest CT (Fig. 3) after a median of 9.3 [9.3–10.8] years of follow-up. Indications for subsequent CCTA were stable chest pain in 77% (*n* = 24), acute chest pain in 13% (*n* = 4), and 10% (*n* = 3) were asymptomatic. Chest CT were performed for lung nodule follow up (17%, *n* = 27), assessment of known or suspected malignancy (41%, *n* = 66), suspected pulmonary embolism (14%, *n* = 22), symptoms of shortness of breath, cough, or chest pain (22%, *n* = 35), trauma (1.9%, *n* = 3), other indications (4%, *n* = 6) or was not documented (2%, *n* = 3).

**Table 1:** Characteristics of patients with normal coronary arteries who had subsequent cardiac CT or chest CT

		Total	Subsequent cardiac CT			Subsequent chest CT		
			No	Yes	*p*	No	Yes	*p*
Number		524	493	31		362	162	
Male		198 (38%)	186 (38%)	12 (39%)	1.000	143 (40%)	55 (34%)	0.265
Age (years)		53 ± 10	53 ± 10	51 ± 6	0.249	52 ± 10	54 ± 9	**0.012**
BMI (kg/m^2^)		30 ± 6	30 ± 6	32 ± 6	0.073	30 ± 6	30 ± 6	0.439
Atrial fibrillation		8 (1.5%)	8 (1.6%)	0	1.000	6 (1.7%)	2 (1.2%)	1.000
Diabetes mellitus		33 (6.3%)	31 (6.3%)	2 (6.5%)	1.000	21 (5.8%)	12 (7.4%)	0.614
Smoking habit	Current smoker	97 (19%)	91 (19%)	6 (19%)	0.989	57 (16%)	40 (25%)	**0.036**
	Former smoker	133 (25%)	125 (25%)	8 (26%)		92 (25%)	41 (26%)	
	Non-smoker	293 (56%)	276 (56%)	17 (55%)		213 (59%)	80 (50%)	
Hypertension		120 (23%)	114 (23%)	6 (19%)	0.769	83 (23%)	37 (23%)	1.000
Family history of CAD		229 (44%)	211 (43%)	18 (58%)	0.151	155 (43%)	74 (46%)	0.561
Total cholesterol (mg/dL)		195 ± 68	195 ± 67	198 ± 74	0.825	193 ± 68	200 ± 66	0.322
HDL cholesterol (mg/dL)		40 ± 28	40 ± 28	40 ± 25	0.941	39 ± 28	43 ± 28	0.215
10-year cardiovascular risk score		10 [6, 15]	10 [6, 16]	10 [8, 14]	0.781	9 [6, 14]	11 [7, 19]	**0.006**

Number and percent (%), mean ± standard deviation, or median [interquartile range]. Cardiovascular risk score calculated using the ASSIGN score

Bold indicates *p* < 0.05

*BMI* body-mass index, *HDL* high-density lipoprotein, *CAD* coronary artery disease

### Subsequent CT imaging

Of the 31 patients who underwent subsequent cardiac CT, 17 (55%) had CCTA alone, 13 (42%) had CCTA and non-contrast CT for coronary artery calcium scoring, and 1 (3%) had non-contrast CT for coronary artery calcium scoring alone. There were no differences in the baseline clinical characteristics of patients who did or did not have a subsequent cardiac CT (Table 1). There was also no difference in the image quality of the baseline CCTA between those who did or did not have a subsequent cardiac CT. This included the frequency of non-diagnostic images (0 (0%) vs 5 (1%), *p* = 1.0), heart rate (61 ± 10 vs 60 ± 10 beats per minute, *p* = 0.063) and the presence of sinus rhythm (30 (97%) vs 478 (97%), *p* = 1.0), which were similar in patients who did or did not have a subsequent cardiac CT. At 10 years, patients who had a subsequent cardiac CT were more likely to be taking preventative medication (10 (32%) vs 51 (10%), *p* = 0001).

Patients who had a subsequent chest CT were older, more likely to be smokers, and had a higher 10-year cardiovascular risk score compared to those who did not have a subsequent chest CT (Table 1). Patients who had a subsequent chest CT were more likely to be taking preventative medications at 10 years (27 (17%) vs 34 (9.4%), *p* = 0.026).

### Development of CAD

**Fig. 4:**
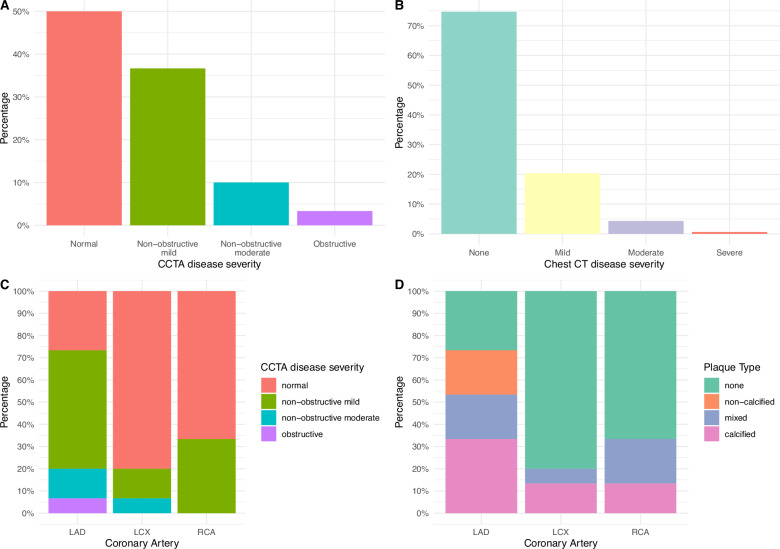
CAD on subsequent CT. Boxplots showing (**A**) the severity of CAD on subsequent coronary CT angiogram, (**B**) the severity of CAD on subsequent chest CT, (**C**) the location of CAD on subsequent coronary CT angiogram, and (**D**) the plaque type identified on subsequent coronary CT angiogram. LAD, left anterior descending coronary artery; LCX, left circumflex coronary artery; RCA, right coronary artery

Of the 31 patients who had a subsequent cardiac CT, CAD had developed in 48% (*n* = 15). Of these patients, one only underwent non-contrast CT for calcium scoring and had a score of zero. On the CCTA scans, normal coronary arteries were identified in 53% (*n* = 16/30) of patients, mild non-obstructive CAD in 36% (*n* = 11/30), moderate non-obstructive CAD in 10% (*n* = 3/30) and obstructive disease in 3% (*n* = 1/30, Fig. 4). The left anterior descending coronary artery was the most common vessel for CAD to have developed in (Fig. 4) and calcified plaque was the most frequently observed plaque type (Fig. 4). Patients who had developed CAD on cardiac CT were more likely to be smokers but had no other differences in the presence of cardiovascular risk factors (Table 2).

**Table 2:** Characteristics of patients with normal coronary arteries on the baseline CCTA who did and did not have CAD on the subsequent CT

		CAD on subsequent cardiac CT			CAD on subsequent chest CT		
		No	Yes	*p*	No	Yes	*P*
Number		16	15		121	41	
Male		7 (44%)	5 (33%)	0.821	42 (35%)	13 (32%)	0.873
Age (years)		51 ± 7	50 ± 6	0.696	53 ± 9	56 ± 9	**0.049**
BMI (kg/m^2^)		32 ± 7	31 ± 5	0.752	30 ± 6	31 ± 7	0.101
Atrial fibrillation		0	0	-	1 (0.8%)	1 (2.4%)	1.000
Diabetes mellitus		1 (6.2%)	1 (6.7%)	1.000	8 (6.6%)	4 (9.8%)	0.749
Smoking habit	Current smoker	0	6 (40%)	**0.014**	26 (22%)	14 (34%)	0.279
	Ex-smoker	6 (38%)	2 (13%)		32 (27%)	9 (22%)	
	Non-smoker	10 (63%)	7 (47%)		62 (52%)	18 (44%)	
Hypertension		4 (25%)	2 (13%)	0.714	28 (23%)	9 (23%)	1.000
Family history of CAD		8 (50%)	10 (67%)	0.565	55 (46%)	19 (46%)	1.000
Total cholesterol (mg/dL)		184 ± 79	213 ± 69	0.276	197 ± 66	209 ± 68	0.309
HDL cholesterol (mg/dL)		33 ± 30	48 ± 16	0.088	44 ± 29	38 ± 26	0.279
Cardiovascular risk score		10 [8, 11]	12 [8, 15]	0.265	10 [7, 16]	15 [9, 21]	**0.002**

Number and percent (%), mean ± standard deviation, or median [interquartile range]. Cardiovascular disease has been calculated using the ASSIGN 10-year cardiovascular risk score

Bold indicates *p* < 0.05

*BMI* body-mass index, *HDL* high-density lipoprotein, *CAD* coronary artery disease

Of the 162 patients who underwent subsequent chest CT, coronary artery calcification was identified on 25% (*n* = 41/162), with mild calcification in 20% (*n* = 33/162), moderate in 4% (*n* = 7/162) and severe in 0.6% (*n* = 1/162, Fig. 4). Patients who had developed CAD identifiable on chest CT had a higher 10-year cardiovascular risk score (15 [9 to 21] vs 10 [7 to 16], *p* = 0.002) and were older (56 ± 9 vs 53 ± 9, *p* = 0.049) than patients who did not have a subsequent chest CT (Table 2).

**Fig. 5:**
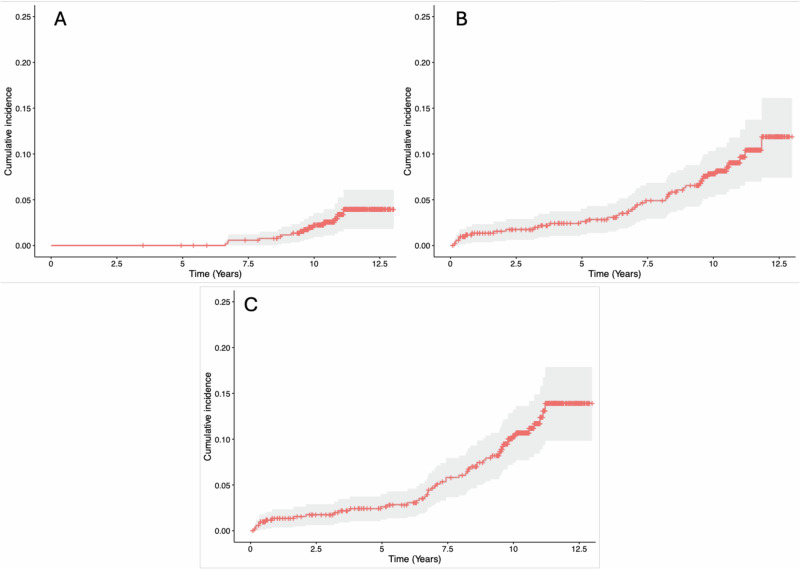
Cumulative time to develop CAD on (**A**) cardiac CT, (**B**) chest CT, and (**C**) all subsequent CT scans

After a median of 10.5 [9.5–11.5] years, 51 (28%) of the 180 patients who had undergone CT had developed CAD. The median time to cardiac CT on which CAD was identifiable was 8.1 [6.9–9.7] years, on chest CT was 7.0 [3.3–8.9] years, and on any CT was 7.4 [5.1–9.5] years (Fig. 5).

### Cardiovascular outcomes

The presence of CAD on subsequent CT was not an independent predictor of the combined outcome of CAD death or non-fatal myocardial infarction or of all-cause mortality, although the number of events was small (Table 3). Patients with CAD identifiable on subsequent CT were more likely to undergo invasive coronary angiography and revascularization compared to patients who did not (Table 3). All revascularization procedures were percutaneous coronary interventions, and no coronary artery bypass graft surgery was performed.

**Table 3:** Clinical outcomes at 10 years in patients with normal coronary arteries on the baseline CCTA who did or did not have CAD identified on subsequent CT

	CAD on subsequent CT		Hazard ratio (95% CI)	*p* value
	No	Yes		
Number	473	51	-	-
CAD death or non-fatal myocardial infarction	6 (1.3)	2 (3.9)	3.12 (0.63, 15.47)	0.163
All-cause mortality	10 (2.1)	3 (5.9)	2.71 (0.74, 9.85)	0.13
Invasive coronary angiography	13 (2.7)	7 (14)	**5.17** **(2.06, 12.96)**	**< 0.001**
Revascularization	1 (0.2)	2 (3.9)	**16.76** **(1.51, 185.45)**	**0.022**

Bold indicates *p* < 0.05

One patient with a normal initial CCTA had no further imaging but presented 10 years later with an ST elevation myocardial infarction and underwent primary PCI. Another patient, also with a normal baseline CCTA, presented with an NSTEMI 8 years later and had developed CAD identifiable on chest CT. A third patient with initially normal CCTA presented 10 years later with stable chest pain. Repeat CCTA at this time revealed obstructive CAD in the proximal left anterior descending coronary artery, and the patient subsequently underwent revascularization. The presence of CAD on subsequent CT was an independent predictor of both the use of invasive coronary angiography (HR 4.94, 95% CI: 1.95, 12.51, *p* < 0.001) and revascularization (HR 19.99, 95% CI: 1.69, 237.1, *p* = 0.018) when adjusted for age and sex.

## Discussion

In this study, we found that a third of patients with normal coronary arteries on CCTA underwent repeat CT imaging that included the heart within 10 years, with 6% undergoing clinically indicated repeat cardiac CT. It was not possible to predict based on baseline cardiovascular risk factors which patients would undergo repeat dedicated cardiac CT. One third of patients with initially normal coronary arteries developed CAD identifiable on repeat CT during the 10-year follow-up period. Patients who developed CAD confirmed on cardiac CT were more likely to be smokers. Patients who developed CAD identifiable on chest CT were more likely to be older and have a higher cardiovascular risk score, although this is likely confounded by the indication. This study highlights the potential need for larger systematic evaluations of CAD development and progression on serial CCTA.

The ability to predict which patients will develop CAD would offer an opportunity to provide personalized targeted therapies to prevent or to delay its initiation. Traditionally, the risk of CAD has been assessed using risk scores. The Framingham Heart Study was pivotal in providing evidence linking risk factors with the development of cardiovascular disease [10]. Multiple risk prediction tools have been created since then with the aim of predicting future risk of cardiovascular events [11]. However, these models reflect population averages and so may underestimate risk in certain subgroups, such as women and younger patients. Consequently, there is a shift towards imaging-based methods to identify and to monitor the presence and severity of CAD. A variety of cardiovascular risk factors are associated with increased prevalence of CAD on CCTA and the presence of high-risk plaque, including age, sex, body mass index, smoking status, and hypertension [1, 12]. In this study, we showed that it was not possible to predict from the baseline clinical characteristics which patients would subsequently develop CAD. Understanding the natural history of CAD development and progression would be invaluable for recommendations regarding optimal time intervals for follow-up imaging and initiation of preventative therapies. We found a median time to develop CAD of 8.1 years in this study. However, this is based on clinically indicated CT rather than systematic assessment, and this may have led to over- or under-estimation of the results. In addition, chest CT can miss non-calcified coronary artery plaque, and further systematic studies with dedicated cardiac CT are warranted.

No studies have previously assessed the development of CAD on CCTA in patients with an initially normal CCTA. The SCOT-HEART trial assessed patients with symptoms of angina due to suspected CAD. In contrast, the Swedish CArdioPulmonary bioImage Study 2 (SCAPIS2, NCT06679777) study will assess serial CCTA in 15,000 asymptomatic subjects, but this represents a low-risk asymptomatic cohort. Using non-contrast CT for calcium scoring, small studies of asymptomatic patients have shown that in patients with a calcium score of zero, between 23 and 25% will develop coronary artery calcification after around 5 years of follow-up [13, 14]. A larger study of 6268 asymptomatic Korean patients found that between 13 and 28% of patients developed coronary artery calcification after 10 years, depending on their cardiovascular risk score [15]. In the Multi-Ethnic Study of Atherosclerosis (MESA) study, which assessed asymptomatic individuals, 36% of patients with a baseline calcium score of zero developed coronary artery calcification with a mean time to detection of 6 ± 3 years [16]. CAC progression has also been assessed in heart transplant patients, with age, donor age, sex, and the presence of ischemic cardiomyopathy all associated with CAC progression [17]. These findings highlight that patients with initially normal coronary arteries remain at risk of developing CAD over time, and it is important to further investigate which patients are at increased risk so that preventative measures can be targeted. In particular, studies assessing serial CCTA with the ability to assess high-risk plaque types such as non-calcified and low attenuation plaque would provide valuable insights into the development and progression of CAD, and the impact of targeted medical therapies on CAD progression [18].

Patients with normal coronary arteries have a good long-term prognosis. In the Coronary CT Angiography Evaluation for Clinical Outcomes: An International Multicenter (CONFIRM) registry, the annual cardiovascular event rate for patients with normal coronary arteries was 0.36% [19]. In the CAC Consortium registry of 66,363 patients, 29,757 patients had a calcium score of zero, and these patients had a low rate of coronary heart disease death and cardiovascular disease death (0.32 and 0.43 per 1000 person years, respectively) [2]. Indeed, a warranty period of 15 years has been suggested for patients with a coronary artery calcium score of zero [20]. Amongst 4864 patients with a calcium score of zero, there was a warranty period with an all-cause mortality rate below 1% of almost 15 years [20]. Similarly, in our study, patients with normal initial CCTA had a good long-term prognosis with a low number of events after 10 years of follow-up. However, in the Heinz Nixdorf Recall study, development of coronary artery calcification was associated with an increased risk of coronary and cardiovascular events [21]. In the Progression of AtheRosclerotic PlAque DetermIned by Computed TomoGraphic Angiography IMaging (PARADIGM) registry, the annualized change in percent atheroma volume, adjusted for the baseline volume, was independently associated with major adverse cardiovascular events (odds ratio 1.23, 95% CI: 1.08, 1.39 per 1 standard deviation increase) [3]. Identifying patients who are at risk of developing CAD may help to identify patients who would benefit from targeted medical therapy.

This study has some limitations that should be acknowledged. First, patients underwent clinically indicated repeat CCTA or chest CT for other indications. The results are therefore impacted by selection bias and confounding by indication, as only patients with symptoms or a reason for chest CT would be included in this study. We would expect that the proportion of patients with CAD identified in this sub-study is likely to be higher than if all patients underwent repeat imaging. Systematic analysis with repeated CCTA in the SCOT-HEART trial would therefore be valuable. The use of preventative medication, such as statin therapy, may have also impacted the development of CAD in these patients. We used the baseline clinical information in this study, but other clinical factors, such as the development of new cardiovascular risk factors. A small number of patients will have moved from Scotland, and their imaging would not have been available in the Scottish National PACS system for inclusion in this study. On chest CT, only calcified coronary artery plaque can be identified, and this may have missed earlier non-calcified plaque, which could have developed in some patients. Non-calcified plaque, particularly low attenuation plaque, may represent a higher risk of CAD, and not including it in our analysis is an important limitation. In addition, motion artifact on chest CT can lead to under- or over-estimation of the presence and severity of CAD. We used a semi-quantitative visual assessment of coronary artery calcification on chest CT, which has good agreement with quantitative metrics such as the Agatston calcium score [22].

In conclusion, in this sub-study, one-third of patients with initially normal CCTA who underwent repeat clinically indicated CT have developed CAD over the follow-up period of 10 years.

## Abbreviations

CI: Confidence intervals
CAD: Coronary artery disease
CCTA: Coronary computed tomography angiography
HR: Hazard ratios
IQR: Interquartile range
SCOT-HEART: Scottish Computed Tomography of the HEART
